# Facile Synthesis with TiO_2_ Xerogel and Urea Enhanced Aniline Aerofloat Degradation Performance of Direct Z-Scheme Heterojunction TiO_2_/g-C_3_N_4_ Composite

**DOI:** 10.3390/ma15103613

**Published:** 2022-05-18

**Authors:** Sipin Zhu, Zhiyong Chen, Chunying Wang, Jiahao Pan, Xianping Luo

**Affiliations:** 1 Faculty of Resource and Environmental Engineering, Jiangxi University of Science and Technology, Ganzhou 341000, China; zhusipin926@163.com (S.Z.); beyond_life@163.com (C.W.); thekid123go@163.com (J.P.); 2 Jiangxi Key Laboratory of Mining & Metallurgy Environmental Pollution Control, Ganzhou 341000, China; 3 Ganfeng Lithium Co., Ltd., Ganzhou 341000, China; 18970142740@163.com

**Keywords:** titanium dioxide, graphite phase carbon nitride, photocatalysis, Aniline Aerofloat

## Abstract

Different TiO_2_/g-C_3_N_4_ (TCN) composites were synthesized by a simple pyrolysis method with TiO_2_ xerogel and urea. The structure and physicochemical properties of TCN were characterized by X-ray diffraction, scanning electron microscope, transmission electron microscope, ultraviolet-visible diffuse reflectance spectrum, X-ray photoelectron spectroscopy, N_2_-adsorption isotherms and electrochemical impedance spectroscopy. Aniline Aerofloat was chosen as a typical degradation-resistant contaminant to investigate the photodegradation activity of TCN under UV irradiation. The results indicated that TCN had higher light absorption intensity, larger specific surface area and smaller particle size compared to pure TiO_2_. Furthermore, TCN had great recycling photocatalytic stability for the photodegradation of Aniline Aerofloat. The photocatalytic activity depends on the synergistic reaction between holes (h^+^) and hydroxyl radicals (·OH). Meanwhile, the direct Z-scheme heterojunction structure of TiO_2_ and g-C_3_N_4_ postpones the recombination of h^+^ and electrons to enhance UV-light photocatalytic activity.

## 1. Introduction

Aniline Aerofloat (dianilinodithiophosphoric acid, AAF, (C_6_H_5_NH)_2_PSSH) has been used frequently as an efficient flotation collecting agent for lead-zinc sulfide ore in China [1]. It was discharged with flotation wastewater into nature although it is highly toxic and contains dithiophosphate and aniline groups [2,3,4]. Moreover, AAF has been hard to degrade thoroughly so far. Some degradation methods have been researched such as sodium hypochlorite oxidation [5], vacuum ultraviolet/ozone [6], chelate precipitation [7], and so on. Thereinto, photocatalytic degradation has great potential to degrade AAF quickly, easily, and effectively.

In the photocatalytic degradation of organic pollution, titanium dioxide (TiO_2_) has been researched extensively in dyes, endocrine disrupters, pesticides, etc., as a photocatalyst [8,9,10]. It deserves more attention even as a commercialized attribute to safety, non-toxicity, stability, high-performance, and low cost [11,12,13]. However, TiO_2_ was limited in practical applications due to its wide bandgap (3.2 eV) and the rapid recombination of electron (e^−^) and hole (h^+^). The construction of TiO_2_/semiconductor heterojunction is an effective method in many ways. Graphene carbon nitride (g-C_3_N_4_) has a narrow bandgap (2.7 eV) with an electronic structure, excellent visible light responding and is easy to prepare in the meantime [14,15]. Therefore, g-C_3_N_4_ was identified as an appropriate and complementary semiconductor to composite TiO_2_/semiconductor heterojunction [16,17,18]. For example, Tan et al. prepared Ti^3+^ self-doped TiO_2_/g-C_3_N_4_ heterojunctions via solid-state chemical reduction, and the material showed better degradation of phenol and production of hydrogen [19]. Wei et al. synthesized mesoporous brookite/anatase TiO_2_/g-C_3_N_4_ heterojunction hollow microspheres that had a more negative conduction band and a wider light absorption range [20]. Mesoporous TiO_2_/g-C_3_N_4_ composite was fabricated by Zhang et al. had about 50 times the removal rate of enrofloxacin than pure g-C_3_N_4_, which attribute to the O–Ti–N bond interface heterojunction between TiO_2_ and g-C_3_N_4_ separated and transferred charge carriers efficiently [21]. To enhance TiO_2_ light response, a uniform mesopore TiO_2_/g-C_3_N_4_ composite with core/shell heterojunction structure was prepared by Xia et al., and the uniform mesoporous g-C_3_N_4_ nanosheets were used to decorate TiO_2_ spheres [22].

The TiO_2_/g-C_3_N_4_ composite was studied deeply by lots of specialists and multitudinous synthesis methods and raw materials were exploited. In the synthesis process of TiO_2_/g-C_3_N_4_, there are many ways to prepare such as adding g-C_3_N_4_ powder in a precursor solution of TiO_2_, depositing TiCl_4_ precursor onto the surface of the g-C_3_N_4_ powder, mixing TiO_2_ powder with melamine in solution, and so on [23,24,25,26].

To our knowledge, even though so many methods were adopted to prepare TiO_2_/g-C_3_N_4_ composite, TiO_2_ xerogel has not been directly mixed with urea as raw materials to synthesize the composite. TiO_2_ xerogel was used rather than commercial TiO_2_ (P25), which could compose better with g-C_3_N_4_ during TiO_2_ catalyst forming. In this work, TiO_2_/g-C_3_N_4_ composite was synthesized by simple pyrolysis with urea and TiO_2_ xerogel, and AAF was selected as the potential pollutant to detect the photocatalytic activities of the prepared composite catalyst. Synthesis conditions including the mass ratio of TiO_2_ to C_3_N_4_, calcination temperature, calcination time, and heating rate were studied detailed. X-ray diffraction, scanning electron microscope, transmission electron microscope, ultraviolet-visible diffuse reflectance spectrum, X-ray photoelectron spectroscopy N_2_-adsorption isotherms and electrochemical impedance spectroscopy were all characterized to analyze the heterojunction structure and physicochemical properties of TiO_2_/g-C_3_N_4_. Furthermore, the stability and mechanism of TiO_2_/g-C_3_N_4_ were also deduced.

## 2. Materials and Methods

### 2.1. Materials

Aniline Aerofloat (AAF, (C_6_H_5_NH)_2_PSSH, 99.5%): Hunan Mingzhu Flotation Reagents Limited Company, Hunan, China. Ethylene glycol (CH_3_CH_2_OH, 99.7%): Tianjin Guangfu Science and Technology Development Co., Ltd., Tianjin, China. Glacial acetic acid (CH_3_COOH, 99.5%): Xilong Scientific Co., Ltd., Guangdong, China. Ammonium oxalate ((NH_4_)C_2_O_4_, 99.8%): Damao Chemical Reagent Factory, Tianjin, China. Methanol (CH_3_OH, 99.9%), tetrabutyl titanate (Ti(OC_4_H_9_)_4_, 98.0%), isopropanol (C_3_H_8_O, 99.7%) and urea ((NH_2_)_2_CO, 99.0%): Sinopharm Chemical Reagent Co., Ltd., Shanghai, China. The reagents above are all used without further purification.

### 2.2. Synthesis of Photocatalysts

#### 2.2.1. Synthesis of TiO_2_ Xerogel

The TiO_2_ xerogel was prepared by the sol-gel method [27,28]. In preparing solutions A and B, respectively, solution A was the mixture consisting of 10 mL ethyl alcohol and 2 mL glacial acetic acid; solution B could be obtained when the 10 mL tetrabutyl titanate was dripped into 15 mL ethyl alcohol slowly at a speed of 0.1 mL·s^−1^ with magnetic stirring. The next step is adding solution A to solution B at a speed of 0.1 mL·s^−1^, the transparent colloid was obtained after 30 min. The transparent colloid was naturally aged for 48 h and dried in the oven at 90 °C for 24 h. Finally, xerogel powder was collected after ground.

#### 2.2.2. Synthesis of TiO_2_/g-C_3_N_4_

**Scheme 1:**
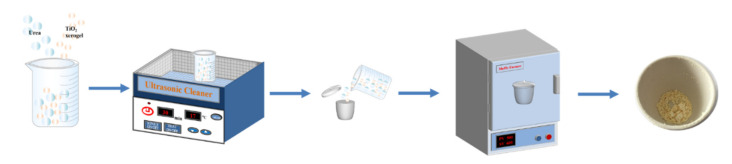
The synthesis of simple TiO_2_/g-C_3_N_4_ by pyrolysis process.

According to previous reports [29,30,31,32], TiO_2_/g-C_3_N_4_ composite photocatalysts were prepared with a simple pyrolysis process. Urea (20 g) and TiO_2_ xerogel powder (2, 1, 0.67, 0.5, 0.4 g) were mixed and vibrated for 30 min with ultrasonic (KQ5200E, 40 KHz, Kunshan Ultrasonic Instruments Co., Ltd., Kunshan, China). The solid mixture was placed into the ceramic crucible with a cover and calcined at different temperatures for several hours at a heating rate with the muffle furnace. The details are illustrated in Scheme 1. The resultant powder which was ground and collected after cooling to an ambient temperature was TiO_2_/g-C_3_N_4_ composite denoted as TCN. TiO_2_/g-C_3_N_4_ composite with a doping mass ratio of x and a heating rate of 5 °C·min^−1^, 550 °C for 6 h is denoted as TCN-U_x_. TiO_2_/g-C_3_N_4_ composite with a doping mass ratio of 20 and a heating rate of 10 °C·min^−1^, y °C for 6 h is denoted as TCN-T_y_. TiO_2_/g-C_3_N_4_ composite with a doping mass ratio of 20 and a heating rate of z °C·min^−1^, 550 °C for 6 h is denoted as TCN-R_z_. TiO_2_/g-C_3_N_4_ composite with a doping mass ratio of 20 and a heating rate of 10 °C·min^−1^, 550 °C for n h is denoted as TCN-H_n_ (x = m_urea_·m_xerogel_^−1^ = 10, 20, 30, 40, 50; y = 450, 500, 550, 600, 650; z = 5, 10, 15, 20, 25; n = 2, 3, 4, 5, 6, 7).

### 2.3. Characterization

X’TRA rotating anode powder X-ray diffraction (XRD, PANalytical B.V., X’Pert powder) with Cu Kα radiation (*λ* = 0.154056 nm) in the diffraction angle range of 2*θ* = 10~90° with 0.02° interval. The sample’s phase and composition were identified by it. The morphology of TCN was exhibited by scanning electron microscope (FE SEM, Tescan, Brno, Czech Republic, MIRA3 LMH). Crystal and element imaging of samples were shown via transmission electron microscope (TEM, FEI, Tecnai Hillsboro, USA, G2-20) connected with a CCD camera from Gatan Company (Pleasanton, CA, USA) and X-ray energy dispersive spectrometer from EDAX Company. Ultraviolet-visible diffuse reflectance spectrum (UV-Vis DRS, Shimadzu, Kyoto, Japan, UV-2600PC) was employed in the range of 200~800 nm with 1 nm interval to obtain the optical absorption spectra of materials, and BaSO_4_ was used as the reference. The Brunauer–Emmett–Teller (BET) surface area and Barrett–Joyner–Halenda (BJH) pore width were analyzed by Micromeritics ASAP 2460 nitrogen adsorption apparatus. X-ray photoelectron spectroscopy (XPS, Thermo, Waltham, MA, USA, ESCALAB 250Xi) spectra were operated with a monochromatic Al Kα radiation source (1486.6 eV) and the binding energies were adjusted with C1s (284.8 eV). Electrochemical impedance spectroscopy (EIS) was analyzed by three-electrode cell electrochemical workstation (CHI-660E, Chinstruments, Shanghai, China) with Pt and saturated calomel electrode as the counter and reference electrode, and 0.1 mol·L^−1^ Na_2_SO_4_ aqueous solution as electrolyte. The sample (10 mg) was dispersed in 1 mL ethylalcohol with ultrasonic for 30 min, and then the mixture was dispersed uniformly on the indium tin oxide (ITO) glass as a working electrode within a 1.0 cm^2^ area.

### 2.4. Photocatalytic Degradation and Analysis

The photocatalytic activity of TCN was explored by degradation of AAF under ultraviolet light provided by a 300 W mercury lamp. All photocatalysis experiments were implemented with the rotary photochemical reaction instrument (XPA-7, Xujiang Electromechanical Plant, Nanjing, China). The catalyst of TCN (30 mg) was dispersed in the AAF solution (50 mL, 100 mg·L^−1^) contained in the quartz tube. The first 30 min of the reaction were in the dark to reach absorption equilibrium, and then they were illuminated for 180 min. After every given time interval, 5 mL of solution was obtained and filtered through a 0.45 μm water filtration membrane to be analyzed by high-performance liquid chromatography (HPLC, 1260 infinity, Agilent, Santa Clara, CA, USA).

The concentration of AAF was tested by HPLC, equipped with eclipse plus C18 (3.5 μm, 4.6 mm × 150 mm) and the temperature of the chromatograph column was 30 °C, The measured wavelength was set as 230 nm according to the full wavelength scanning by ultraviolet-visible spectrophotometer (T6 New century, Beijing Persee General Co., Ltd., Beijing, China). The mobile phase consists of water and methanol (*v*/*v* = 60:40) and flows at 1 mL·min^−1^. The degradation efficiency was calculated by C_t_/C_0_, in which C_0_ and C_t_ were the concentration of AAF at the beginning and a certain time.

At the same time, the catalyst was reused 4 times under the same conditions to investigate the stability and the prospects for practicality in industrial. Reactive active species hole (h^+^) and hydroxyl radical (·OH) in the photocatalytic degradation process were analyzed by quenching agent ammonium oxalate and isopropanol experiments.

## 3. Results and Discussion

### 3.1. Morphology Analysis

**Figure 1:**
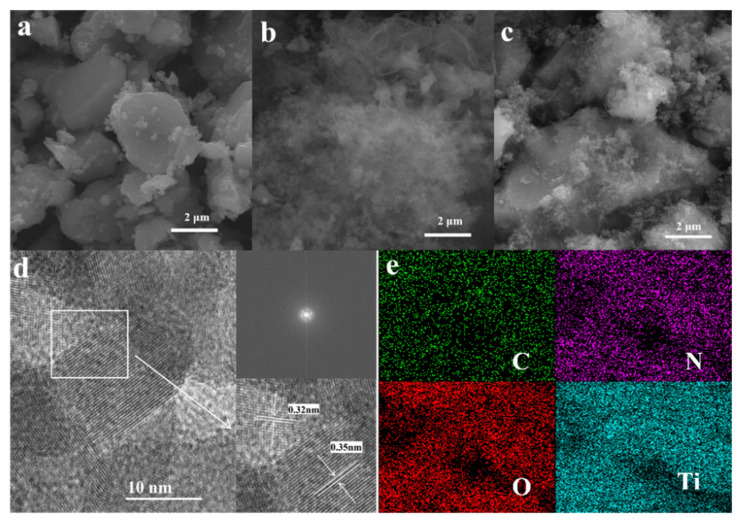
SEM images of (**a**) pure TiO_2_, (**b**) g-C_3_N_4_, and (**c**) TCN-U_20_; (**d**) TEM image, (**e**) mapping images of TCN-U_20_.

SEM images of pure TiO_2_, g-C_3_N_4_, TCN-U_20_ and TCN-T_y_ shown in Figure 1a–c and Figure S1 provided that TiO_2_ and g-C_3_N_4_ were composed successfully. As we can see, the TCN composite combined with the structure of the lamellae g-C_3_N_4_ stack on bulk TiO_2_. The thickness and density of g-C_3_N_4_ lamellae increase, respectively, which made the poriness and active positions more with the doping ratio and calcination temperature higher. Nevertheless, overmuch g-C_3_N_4_ lamellae would impede UV-light absorption of TiO_2_ which was covered and thus weaken the photocatalytic activity of CNT. Meanwhile, the TEM image of TCN-U_20_ shown in Figure 1d suggested the lattice spaces of 0.350 nm and 0.320 nm correspond to the plane (101) of TiO_2_ and the plane (002) of the g-C_3_N_4_ [33,34]. The results of mapping images and EDS spectrum in Figure 1e showed the percent of C, N, O and Ti. The atom percent of C and N is approximately 0.73~0.89 conforming to 0.75 that the ratio of C and N in g-C_3_N_4_. Because of the oxygen in the air, the atom percent of Ti and O is approximately 0.30~0.31, which is far less than 0.5 that the ratio of Ti and O in TiO_2_. The results suggested doping of g-C_3_N_4_ is successful and the formation of the heterojunction between TiO_2_ and g-C_3_N_4_.

### 3.2. XRD Analysis

**Figure 2:**
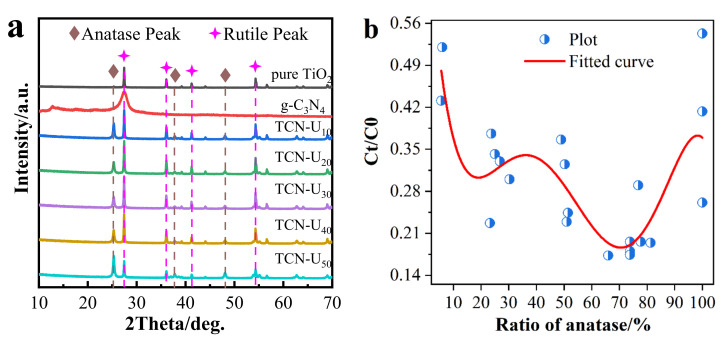
(**a**) XRD patterns of pure TiO_2_, g-C_3_N_4_ and TCN-U_x_ and (**b**) fitted curve between photocatalytic degradation efficiency of AAF and ratio of TiO_2_ anatase.

The crystal phases of TCN, pure g-C_3_N_4_ and TiO_2_ are exhibited in Figure 2a and Figure S2. Diffraction peaks at 25.3°, 37.8° and 48.0° were pointed to (101), (004) and (200) of anatase TiO_2_ (JCPDS 21-1272). Peaks at 27.4°, 36.1°, 41.2° and 54.3° are pointed to (110), (101), (111) and (211) of rutile TiO_2_ (JCPDS 21-1276), and peaks at 12.9° and 27.7° correspond to (100) in the in-plane repetitive unit of tri-s-triazine and (002) attributed to interlayer stacking of g-C_3_N_4_ (JCPDS 87-1526) [35,36,37]. Almost all TCN samples were mixed with the rutile phase and anatase phase of TiO_2_. Observing all XRD patterns of TCN, it is indistinguishable if the peak at 27.7° belongs to the phase of g-C_3_N_4_ due to low loading and overlapped peaks [38]. The calculated crystalline phase ratio and cell parameters of TCN are shown in Table 1 and Table S1. The growth of the TiO_2_ rutile phase was suppressed with the doping of g-C_3_N_4_, and the ratio was lowest when the doping ratio was 20. Similarly, the other results can be seen in Table S1. The growth of the TiO_2_ rutile phase was also suppressed with the heating rate, and the ratio was lowest when the heating rate was 10, and the growths of the TiO_2_ rutile phase were both always promoted with the temperature and heat preservation hour. Suggesting the changes of diffraction peaks were caused by the transformation between TiO_2_ anatase and rutile and the growth of crystal particles. Excellent mixed ratios of TiO_2_ anatase and rutile when the weight percent of the anatase phase was 76.8 or 73.8 and the rutile phase was 23.2 or 26.2 demonstrated excellent photocatalytic activity [39,40]. The change in lattice size was caused by the formation of a heterojunction between g-C_3_N_4_ and TiO_2_ [33]. Obviously, the crystal phase ratio of TiO_2_ anatase and rutile changed with TCN preparation conditions. To clear the relationship between TCN degradation efficiency of AAF and the ratio of TiO_2_ anatase, the degradation efficiency plots were fitted with a curve as shown in Figure 2b. The fittted curve was obtained by the power function with six and the correlation coefficient R-square is 0.618. So, the change of crystal phase ratio not only decided cell size but also affected the photocatalytic activity significantly [41].

### 3.3. UV-Vis DRS Analysis

**Figure 3:**
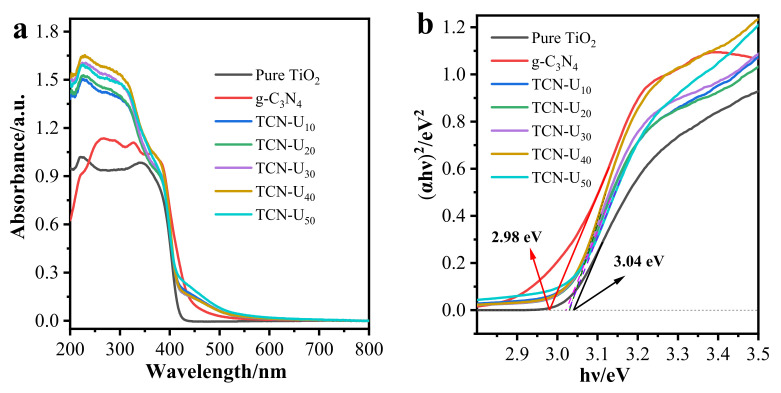
UV-Vis DRS spectra (**a**) and bandgap calculation spectra (**b**) of pure TiO_2_, g-C_3_N_4_ and TCN-U_x_.

The ultraviolet light absorption intensity of all TCN samples was much stronger than pure g-C_3_N_4_ and TiO_2_ as shown in Figure 3a and Figure S3a–c suggested the doped of g-C_3_N_4_ enhanced the UV-light absorption intensity significantly. Furthermore, the bandgap was calculated by the Kubelka–Munk function Equation (1) as follows:

(1) $$\alpha h\nu =A{\left(h\nu -E_{g}\right)}^{2}$$

where *α*, *h*, *ν*, *A*, and *E*_g_ was absorption coefficient, Planck constant, light frequency, a constant, and optical bandgap energy, respectively. The bandgap calculation spectra shown in Figure 3b and Figure S3d–f and the results indicated that different preparation conditions influenced the bandgaps to different degrees. Combined with the XRD results, it can be inferred that the change in UV-light absorption intensity and bandgap were related to the formation of the TiO_2_/g-C_3_N_4_ heterojunction and TiO_2_ phase transformation between anatase and rutile [42,43,44].

### 3.4. S_BET_ and Porosity Analysis

**Figure 4:**
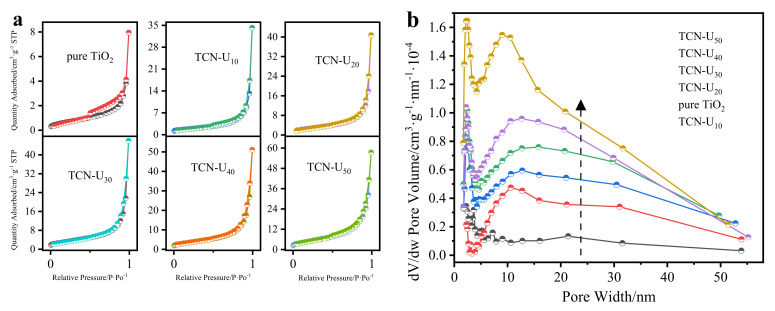
N_2_ adsorption-desorption isotherms (**a**) and pore size distribution (**b**) of pure TiO_2_, g-C_3_N_4_ and TCN-U_x_.

N_2_ adsorption-desorption isotherms and pore size distribution of TCN-U_x_ and pure TiO_2_ showed in Figure 4. The BET fitted curve and specific surface area data of TCN-U_x_ are exhibited in Figure S4, Table 2 and Table S2. Compared with pure TiO_2_, *S*_BET_ of TCN increased 3–7 times after g-C_3_N_4_ doping. All samples showed the typical IV isotherm with H1 hysteresis loops reflecting the existence of homogeneous mesoporous. The hysteresis loops were caused by capillary condensation in the relative pressure (P/P_0_) range of about 0.5–1.0. The results suggested that TCN has a bigger specific surface area and smaller pore size than pure TiO_2_. TCN performed high photocatalytic activity demonstrated by a bigger specific surface area, more abundant mesoporous structure, and more adsorption sites [39]. The increase of S_BET_ was attributed to covered g-C_3_N_4_ increased with doping ratio, and growth of TiO_2_ anatase with heating rate sped within range of 10 °C/min. The decrease of SBET was due to the growth of TiO_2_ rutile with a heating rate speed from 10 to 25 °C/min, calcination temperature raised from 450 to 650 °C, and heating preservation hour increasing from 2 to 7 h. The results were consistent with the XRD and SEM characterization of the samples.

### 3.5. Photodegradation Experiment

**Figure 5:**
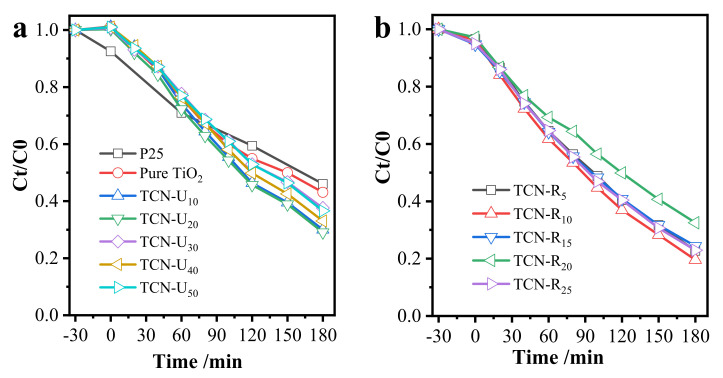

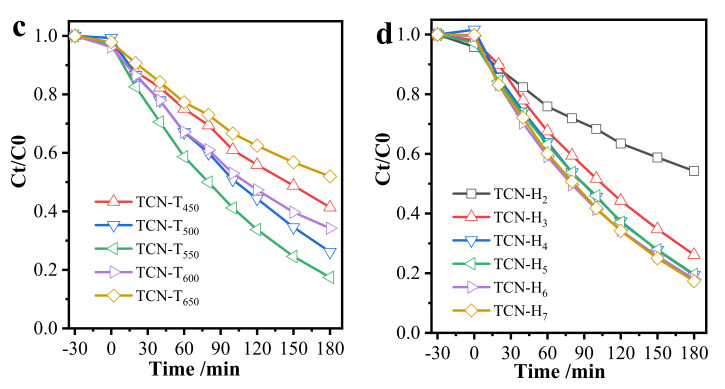
Activity of (**a**) TCN-U_x_, (**b**) TCN-R_z_, (**c**) TCN-T_y_, (**d**) TCN-H_n_ on the photocatalytic degradation of AAF.

The photocatalytic degradation of AAF by TCN (Figure 5) was always higher than by commercial TiO_2_ (P25) and pure TiO_2_ prepared, which might be attributed to the formation of the TiO_2_/g-C_3_N_4_ heterojunction [29,44,45,46,47]. The TCN samples with a 20 doping ratio, 10 °C·min^−1^, 550 °C, 6 h in the same three other conditions showed the optimum photocatalytic degradation efficiency.

The performance of photocatalytic degradation could be attributed to these reasons as results of characterization showed: the bigger specific surface area and more active positions with the doping ratio of g-C_3_N_4_ compared with pure TiO_2_, transforming from urea to g-C_3_N_4_ smoother with the heating rate, completeness, and crystallinity of TiO_2_/g-C_3_N_4_ composite better with the calcination temperature and heating preservation hour. However, the excessive g-C_3_N_4_ would obstruct the UV-light absorption to TiO_2_ with the further increase of doping ratio. The transformation process was too fast to attach uniformly to the TiO_2_ surface resulting in stacking, and TCN got heavily agglomerated with the further rise in calcination temperature. Moreover, a suitable ratio of TiO_2_ rutile phases and anatase phases is a vital contribution to promoting photocatalytic degradation efficiency [40,41,48]. On the whole, the calcination temperature has the greatest influence on the photocatalytic activity of aniline aerofloat degradation, followed by the doping ratio, heat preservation hour and heating rate have the least influence in a certain range.

**Figure 6:**
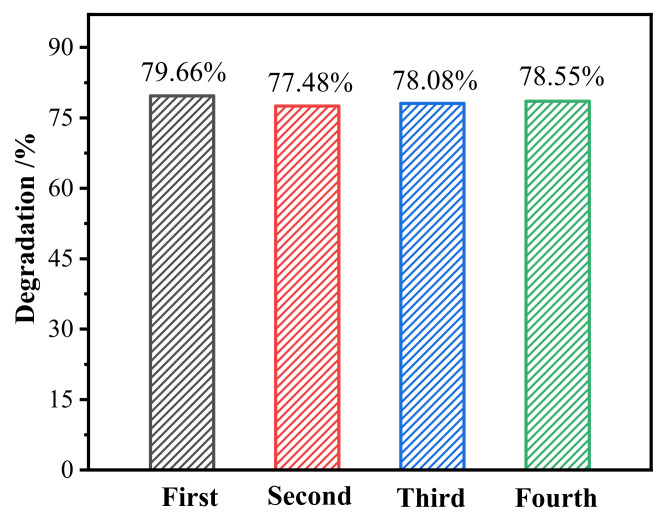
Activity of recycling test of TCN-T_550_ for photocatalytic degradation of AAF.

The stability of the photocatalyst is an important index to estimate the photocatalytic activity so it is significant to study the recycling of the TiO_2_/g-C_3_N_4_ composite. At room temperature, TCN-T_550_ (30 mg) was added to a 50 mL AAF solution (100 mg·L^−1^). The catalyst used was collected and reused in the next degradation experiment. The results of reusing catalyst photocatalytic activity were showed in Figure 6. After four times of recycling, the TiO_2_/g-C_3_N_4_ composite photocatalytic degradation efficiency of AAF was always steadily high, indicating that the TiO_2_/g-C_3_N_4_ composite is a kind of potential photocatalyst for practical engineering application.

### 3.6. XPS Analysis

**Figure 7:**
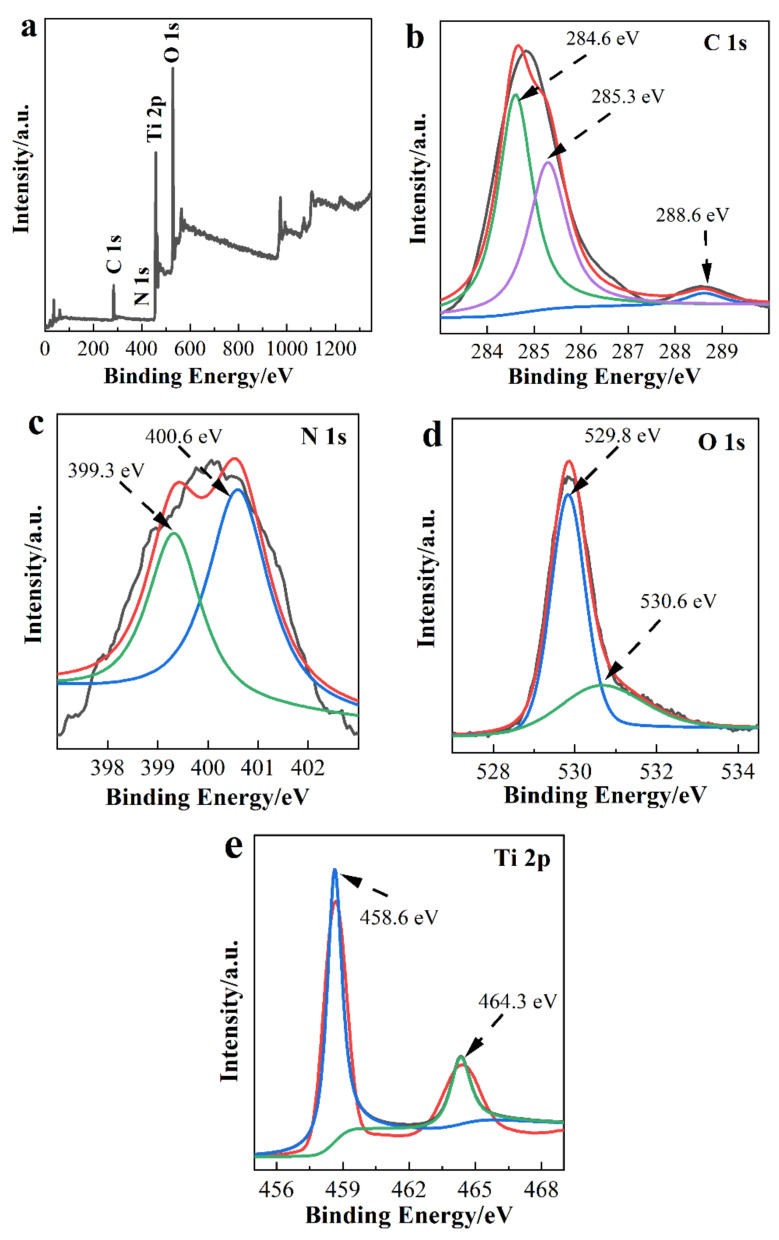
XPS of survey (**a**), C1s (**b**), N1s (**c**), O1s (**d**) and Ti2p (**e**) scans of TCN-T_550_.

The surface chemical compositions of TCN-T_550_ were tested by XPS and the results shown in Figure 7 show survey, C1s, N1s, O1s and Ti2p scans. The peaks at 284.6 eV, 285.3 eV, and 288.6 eV shown in Figure 7b correspond to the sp^2^ C–C, C=, N–C=N bonds [49]. Meanwhile, the binding energies of 399.3 eV and 400.6 eV shown in Figure 7c correspond to the sp^3^ N-(C)_3_ and interstitial N, which indicated the existence of g-C_3_N_4_ and composite structure [8,50]. Binding energies in 529.8 eV and 530.6 eV shown in Figure 7d corresponds to Ti–O band and surface absorbed –OH [44], and binding energies in 458.6 eV and 464.3 eV shown in Figure 7e were attributed to Ti 2p_3/2_ and Ti 2p_1/2_ [51], which directly testified TiO_2_. Furthermore, there no peaks of C–O, N–O, Ti–C, or Ti–N observed indicating that TiO_2_ and g-C_3_N_4_ were composed with molecule doping rather than lattice replace.

### 3.7. Electrochemical Analysis

**Figure 8:**
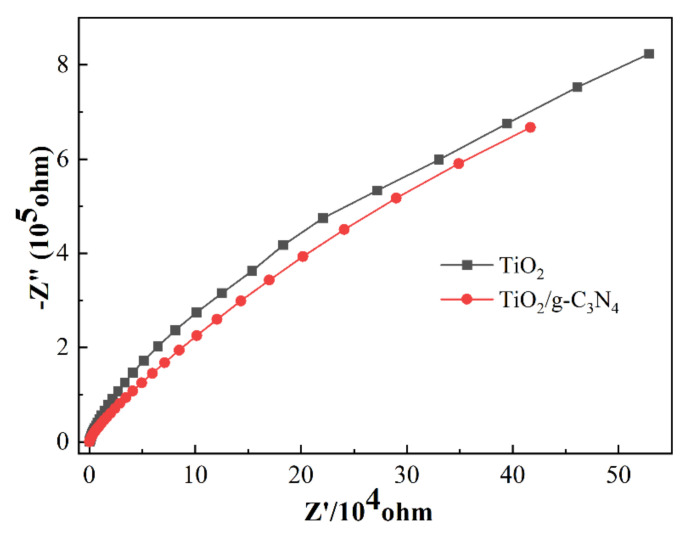
The EIS of pure TiO_2_ and TCN-T_550_.

The electrochemical impedance spectroscopy (EIS) of pure TiO_2_ and TCN-T_550_ shown in Figure 8 indicated that e^−^-h^+^ pair separation-effective of TCN-T_550_ is more efficient than TiO_2_ [49].

### 3.8. Mechanism of Composite

#### 3.8.1. Reactive Active Species of Photocatalyst

**Figure 9:**
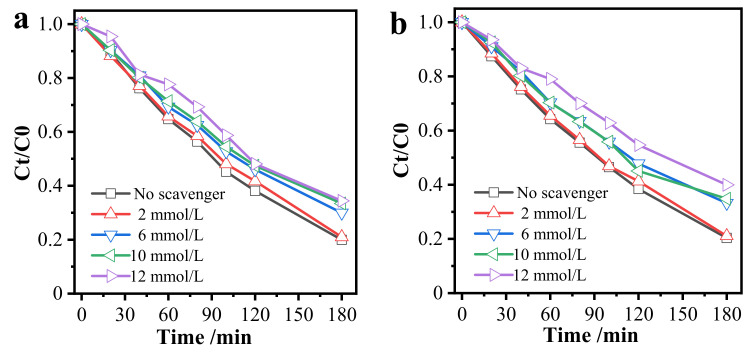
Effect of TCN-T_550_ for photocatalytic of AAF degradation with different concentration quenching agents: (**a**) ammonium oxalate, (**b**) isopropanol.

Research of photocatalysts’ reactive active species in the photocatalytic degradation process is the fundamental research of photocatalytic mechanism. As we all know, ammonium oxalate is the quenching agent of hole (h^+^), and isopropanol is the quenching agent of hydroxyl radical (**·**OH). The results are shown in Figure 9.

The photocatalytic degradation of AAF efficiency declined gradually with the increasing concentration of ammonium oxalate and isopropanol shown in Figure 9. The result illustrates the oxidation action of h^+^ and ·OH in the photocatalytic degradation process. Meanwhile, the effect of ammonium oxalate and isopropanol on the photocatalytic degradation efficiency is similarly judged from the degradation efficiency decrement. In conclusion, the h^+^ and **·**OH, which were both photocatalysts reactive active species in the photocatalytic degradation process, react synergistically in the photocatalytic reaction system.

#### 3.8.2. Mechanism Conjecture of Photodegradation

**Figure 10:**
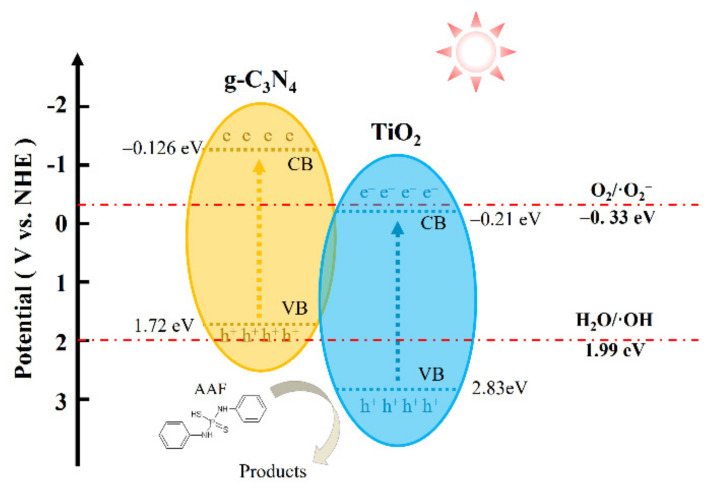
The possible photodegradation mechanism process of TiO_2_/g-C_3_N_4_.

The bandgap energy of TiO_2_ and g-C_3_N_4_ were 3.04 eV and 2.98 eV, which is based on UV-Vis DRS results. The valence band (VB) and conduction band (CB) edge potentials were calculated by the following Equations (2) and (3) [52]:

(2) $${Ε}_{\mathrm{VB}}{=\chi -Ε}^{e}+\frac{1}{2}{Ε}_{g}$$

(3) $${Ε}_{\mathrm{CB}}{=Ε}_{\mathrm{VB}}{-Ε}_{g}$$

where *E*_VB_, *E*_CB_, *χ*, *E*^e^ and *E*_g_ were the VB edge potential, the CB edge potential, the geometric mean of Mulliken electronegativity [53], and the energy of electrons on the hydrogen scale (~4.5 eV, vs. NHE) and optical bandgap energy. The value of *χ* of TiO_2_ and g-C_3_N_4_ were 5.81 eV [54] and 4.73 eV [55]. The *E*_VB_ of TiO_2_ and g-C_3_N_4_ were 2.83 eV and 1.72 eV, and the *E*_CB_ of TiO_2_ and g-C_3_N_4_ were calculated as –0.21 eV and −1.26 eV, respectively. The VB potential of TiO_2_ (2.83 eV) is more positive than H_2_O/**·**OH (+1.99 eV vs. NHE), and the CB potential of g-C_3_N_4_ is more negative than O_2_/**·**O_2_^−^ (−0.33 eV vs. NHE) [56], so there was an oxidation reaction in the VB of TiO_2_ and reduction reaction in the CB of g-C_3_N_4_. This result and the results of photocatalysts reactive active species confirmed each other. TiO_2_ and g-C_3_N_4_ might form type-II or direct Z-scheme heterojunction. If it belongs to type-II heterojunction, e^−^ on the CB of g-C_3_N_4_ would transfer to the CB of TiO_2_ and h^+^ on the VB of TiO_2_ would transfer to the VB of g-C_3_N_4_. However, the VB potential of g-C_3_N_4_ (1.72 eV) is more negative than H_2_O/**·**OH (+1.99 eV vs. NHE) and cannot produce **·**OH which was the active species. The conclusion is contradictory to the result of the quenching experiment. So, the possible photodegradation mechanism process was conjectured as a direct Z-scheme heterojunction shown in Figure 10 [57] and following Equations (4)–(8):

TiO_2_/g-C_3_N_4_ + hν → TiO_2_ (e^−^, h^+^)/g-C_3_N_4_ (e^−^, h^+^) (4)

TiO_2_ (e^−^, h^+^)/g-C_3_N_4_ (e^−^, h^+^) → TiO_2_ (h^+^) + g-C_3_N_4_ (e^−^) (5)

TiO_2_ (h^+^) + H_2_O → **·**OH + H**·** (6)

g-C_3_N_4_ (e^−^) + O_2_ → **·**O_2_^−^ (7)

**·**OH/TiO_2_ (h^+^)/**·**O_2_^−^ + Aniline Aerofloat → products (8)

Under light irradiation excitation, e^−^ in transfers from CB to VB of TiO_2_ and g-C_3_N_4_ severally, and there is h^+^ left in CB. Meanwhile, e^−^ of TiO_2_ combines with h^+^ of g-C_3_N_4_ on the VB of g-C_3_N_4_. Next h^+^ of TiO_2_ oxidizes the H_2_O molecule into **·**OH and H**·** and e^−^ of g-C_3_N_4_ reduces the O_2_ molecule into **·**O_2_^−^. Then, **·**OH, h^+^ and **·**O_2_^−^, which all have oxidizability, can oxidize the Aniline Aerofloat molecule into degraded products.

## 4. Conclusions

TiO_2_/g-C_3_N_4_ composites with different doping ratios, heating rates, calcination temperatures and heating preservation hours were synthesized successfully by a simple pyrolysis method with the raw material of TiO_2_ xerogel and urea. TCN samples with a 20 doping ratio, 10 °C·min^−1^, 550 °C, 6 h in the same three other conditions showed the optimum photocatalytic degradation efficiency. The excellent activity was attributed to the appropriate crystal phase ratio of TiO_2_ rutile phases and anatase phases, high UV-light absorption intensity, big specific surface area, and formation of the TiO_2_/g-C_3_N_4_ heterojunction. In addition, the TiO_2_/g-C_3_N_4_ heterojunction could postpone the recombination of h^+^ and e^−^ to extend the reaction time and enhance the photodegradation efficiency. Furthermore, TiO_2_/g-C_3_N_4_ composites have great photodegradation stability for AAF after four times.

## Figures and Tables

**Table 1:** Crystalline phase ratio and cell parameters of pure TiO_2_ and TCN-U_x_.

Sample	Anatase/wt%	Rutile/wt%	*a* = *b*/nm	*c*/nm
Pure TiO_2_	5.6	94.4	1.42442	2.08097
10:1	30.3	69.7	1.30545	1.99599
20:1	76.8	23.2	0.80742	1.74158
30:1	23.7	76.3	1.05978	2.33788
40:1	26.8	73.2	1.19316	2.29493
50:1	48.9	51.1	1.10269	2.11718

**Table 2:** Specific surface area of pure TiO_2_ and U_x_T_550_R_5_H_6_-TCN.

Sample	g-C_3_N_4_	Pure TiO_2_	10:1	20:1	30:1	40:1	50:1
Surface area(m^2^·g^−1^)	121.0	2.5	7.3	9.7	11.7	13.1	18.1

## Data Availability

Not applicable.

## Supplementary Materials

The following supporting information can be downloaded at: https://www.mdpi.com/article/10.3390/ma15103613/s1, Figure S1: SEM images at 20,000 times of TCN-T_y_: (a) T_450_; (b) T_500_; (c) T_550_; (d) T_600_; (e) T_650_ and (f) EDS spectrum of TCN-U_20_; Figure S2: XRD patterns of TCN: (a) TCN-R_z_, (b) TCN-T_y_, (c) TCN-H_n_; Figure S3: UV-Vis DRS spectra of U_20_T_550_R_z_H_6_-CNT (a,d), TCN-T_y_ (b,e), and TCN-H_n_ (c,f); Figure S4: Fitted curve of BET of TCN-Ux; Table S1: Crystalline phase ratio and cell parameters of TCN-T_y_, TCN-Rz and TCN-_n_; Table S2: Specific surface area of CNT-T_y_, TCN-R_z_ and TCN- H_n_.
