# Supplementary material for: Facile Synthesis with TiO2 Xerogel and Urea Enhanced Aniline Aerofloat Degradation Performance of Direct Z-Scheme Heterojunction TiO2/g-C3N4 Composite
Source: Materials (Basel). 2022 May 18;15(10):3613. doi: 10.3390/ma15103613 (PMC9145884; doi:10.3390/ma15103613)
Supplement: Supplementary file 1 [file materials-15-03613-s001.zip › materials-1665995-supplementary.pdf]

Supporting Information

Facile synthesis with TiO_2 xerogel and enhanced Aniline Aerofloat degradation performance of direct Z-scheme heterojunction TiO_2/g-C_3N_4 composite

Sipin Zhu[1,2], Zhiyong Chen[3], Chunying Wang[1,2], Jiahao Pan[1,2], Xianping Luo[1,2,*]

[1] Faculty of Resource and Environmental Engineering, Jiangxi University of Science and Technology, Ganzhou 341000, China

[2] Jiangxi Key Laboratory of Mining & Metallurgy Environmental Pollution Control, Ganzhou

[3] Ganfeng Lithium Co. Ltd., Ganzhou 341000, China

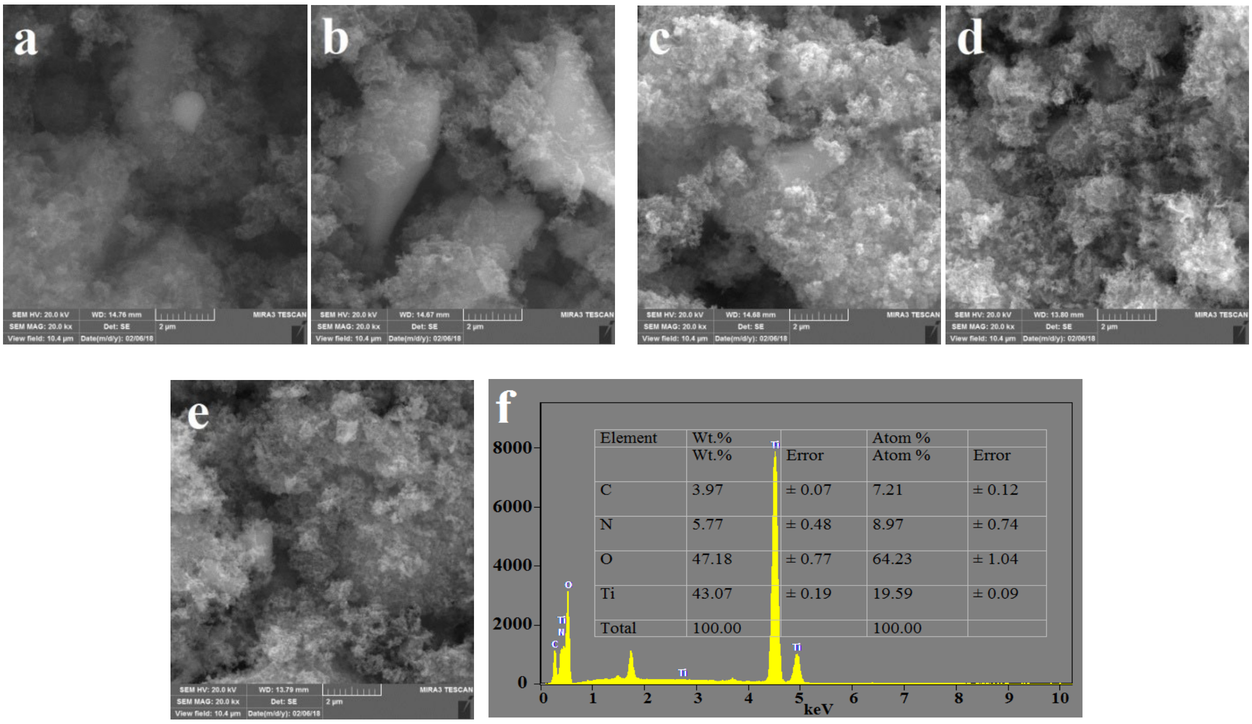

Figure S1. SEM images at 20,000 times of TCN-T_y: (a) T_{450}; (b) T_{500}; (c) T_{550}; (d) T_{600}; (e) T_{650} and (f) EDS spectrum of TCN-U_{20}.

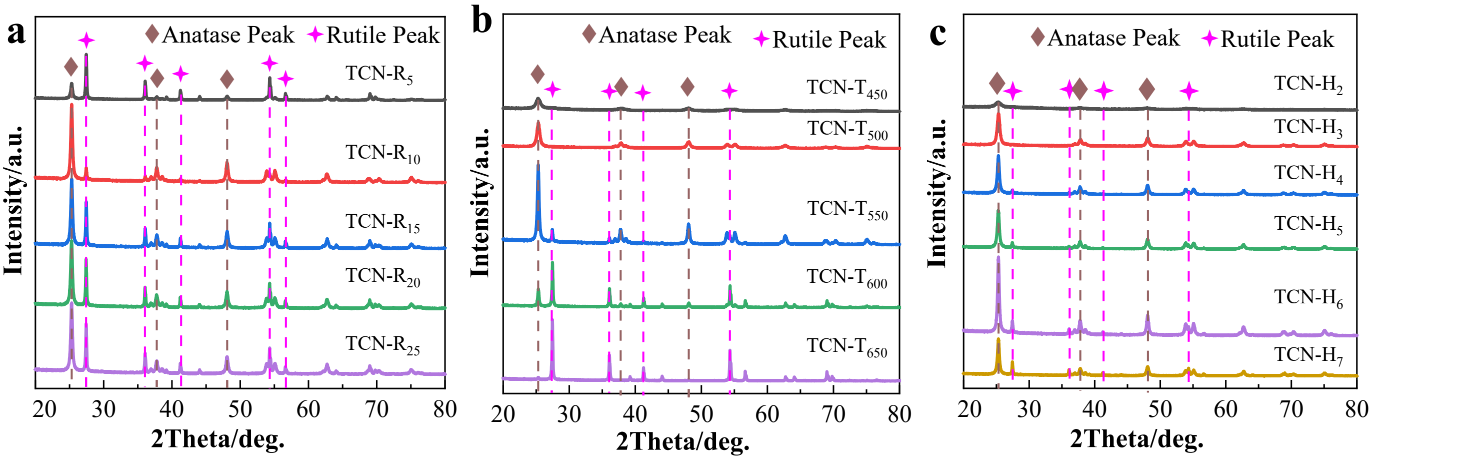

Figure S2. XRD patterns of TCN: (a) TCN-R_z, (b) TCN-T_y, and (c) TCN-H_n.

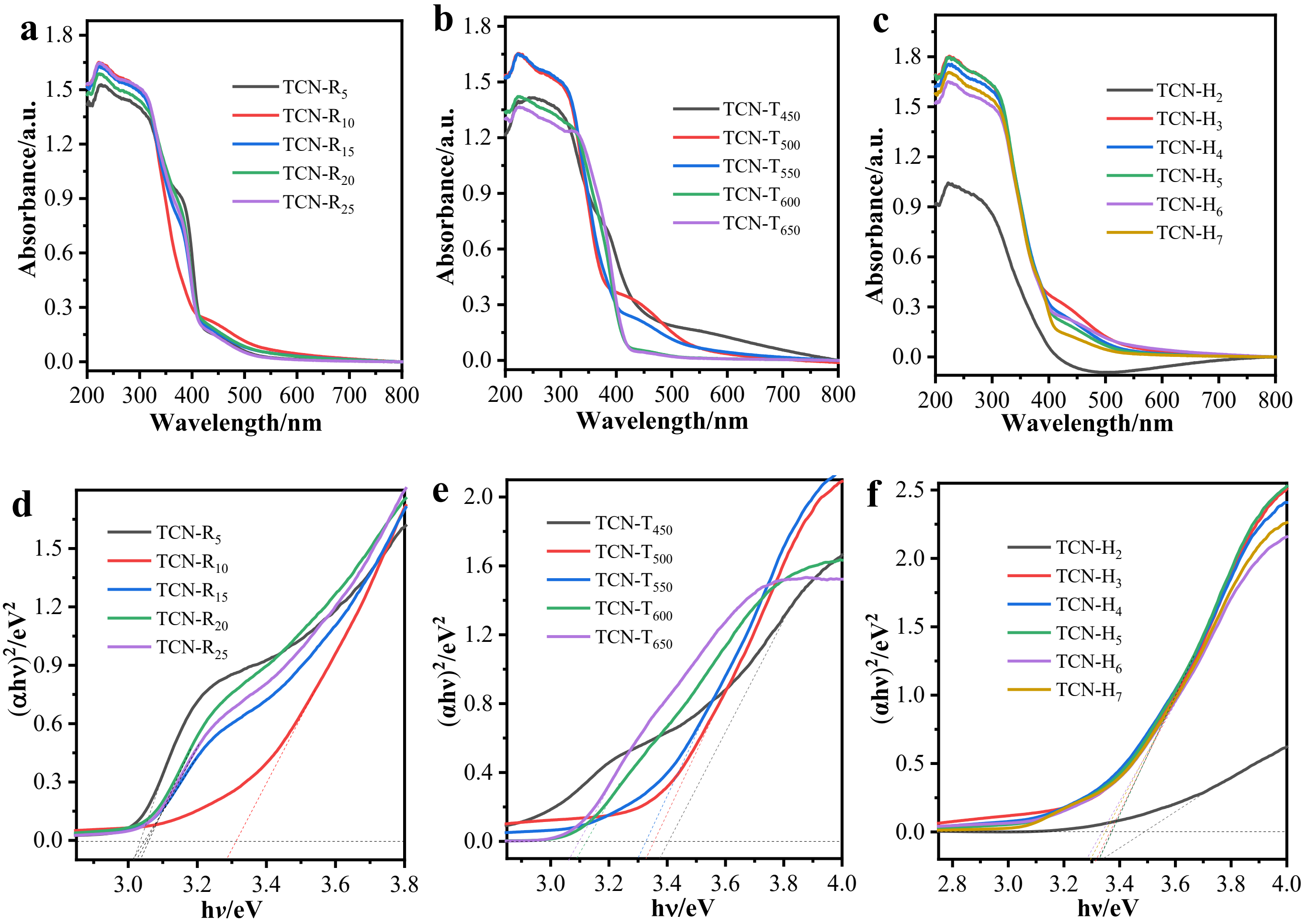

Figure S3. UV-Vis DRS spectra of (a, d) TCN-Rz, (b, e) TCN-T_y, and (c, f) TCN-H_n.

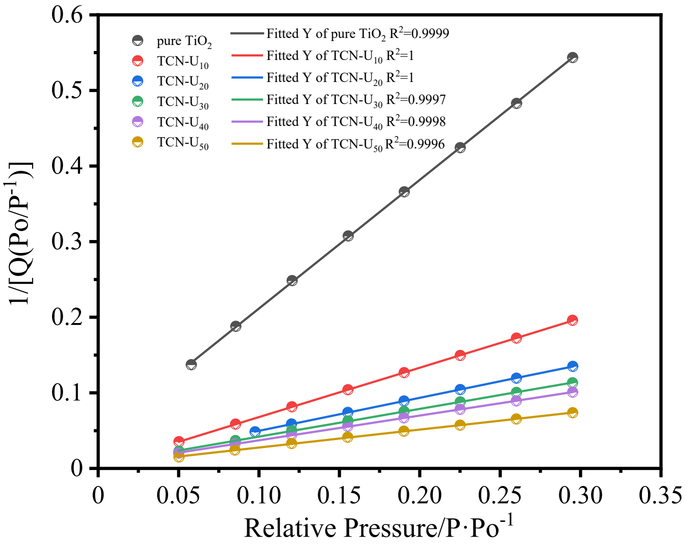

Figure S4. Fitted curve of BET of TCN-U_x

Table S1. Crystalline phase ratio and cell parameters of CNT-T_y, TCN-R_z and TCN-H_n.

Factors	Samples	Anatase/wt%	Rutile/wt%	a=b/nm	c/nm
Heating Rate	5 °C/min	23.2	76.8	0.80742	1.74158
TCN-R_z	10 °C/min	73.8	26.2	1.17464	2.51029
	15 °C/min	51.4	48.6	1.22828	2.67937
	20 °C/min	50.3	49.7	1.18717	2.96092
	25 °C/min	51.0	49.0	0.84848	1.84104
Temperature	450 °C	100	0	0.83034	1.67071
TCN-T_y	500 °C	100	0	0.91318	1.84950
	550 °C	73.8	26.2	1.17464	2.51029
	600 °C	25.0	75.0	0.97731	2.02835
	650 °C	6.0	94.0	0.97763	2.05398
Heat Preservation Hour	2 h	100	0	0.80751	7.07966
	3 h	100	0	1.07155	2.70974
TCN-H_n	4 h	81.3	18.7	1.20373	2.59605
	5 h	77.7	22.3	1.07155	2.33062
	6 h	73.8	26.2	1.17464	2.51029
	7 h	66.0	34.0	1.15187	2.24708

Table S2. Specific surface area of TCN-T_y, TCN-R_z and TCN-H_n.

TCN-R_z	specific surface area ($m^2 \cdot g^{-1}$)	TCN-T_y	specific surface area ($m^2 \cdot g^{-1}$)	TCN-H_n	specific surface area ($m^2 \cdot g^{-1}$)
5 °C/min	9.7	450 °C	101.3	2 h	85.2
10 °C/min	19.3	500 °C	62.1	3 h	63.1
15 °C/min	13.2	550 °C	19.3	4 h	3.8
20 °C/min	12.1	600 °C	6.0	5 h	23.1
25 °C/min	14.1	650 °C	3.5	6 h	19.3
				7 h	20.1
